# Strongyloidiasis in Man 75 Years after Initial Exposure

**DOI:** 10.3201/eid1705.100490

**Published:** 2011-05

**Authors:** Virginie Prendki, Pierre Fenaux, Rémy Durand, Marc Thellier, Olivier Bouchaud

**Affiliations:** Author affiliations: Hôpital Jean Verdier, Bondy, France (V. Prendki); Hôpital Avicenne, Bobingy, France (P. Fenaux, R. Durand, O. Bouchaud);; Université Léonard de Vinci, Bobigny (R. Durand, O. Bouchaud);; Hôpital La Pitié Salpêtrière, Paris, France (M. Theillier)

**Keywords:** strongyloidiasis, Strongyloides stercoralis, parasites, nematode, long latency, hypereosinophilia, letter

**To the Editor:** Strongyloidiasis, caused by the roundworm *Strongyloides stercoralis,* affects 100–200 million persons worldwide (*1*) and is endemic to Southeast Asia, sub-Saharan Africa, Latin America, and the southeastern United States (*2*). Endogenous autoinfection enables this nematode to develop into its host, which leads to the persistence of chronic infection several decades after a person has left a disease-endemic area (*3*). We encountered a case of prolonged strongyloidiasis with an infection going back >75 years.

An 83-year-old man who lived in Paris and had no medical history sought treatment for fatigue and weight loss. Results of a clinical examination were normal. Laboratory investigations showed mild hyperleukocytosis (12 × 10^9^ cells/L) with hypereosinophilia (2.4 × 10^9^ cells/L), which was observed for >3 months. Results of stool examinations were negative for parasites. Because of the patient’s poor condition, cancer or hematologic malignancy were suspected, but results of various examinations, including a computed tomography scan of the body and bone marrow aspiration, were normal.

After several weeks, the patient disclosed that he had spent a few years in Vietnam >75 years ago. The only other travel abroad reported by the patient was a 10-day stay in a tourist hotel in the Canary Islands 15 years before this illness. At this point in his assessment, results of serologic testing were positive for *Strongyloides* spp., and a new stool examination showed *Strongyloides* larvae. Serologic test results were negative for human T-cell lymphotropic virus type 1. The patient received 2 doses of 12 mg of ivermectin within 15 days and fully recovered. Hypereosinophilia and *Strongyloides* larvae in feces disappeared.

*S. stercoralis* roundworms are ubiquitous intestinal parasites, endemic to tropical and subtropical regions. The larvae can develop into filariform larvae, which can penetrate the human skin and migrate through circulation to the lungs before settling in the intestine. In the human host, adult parasites may be generated by parthenogenesis in the mucosa of the small intestine. The resultant larvae can also penetrate the skin or the intestinal mucosa to establish a cycle of repeated endogenous reinfection. The parasite may then cause a long-lived autoinfection in the host, leading to chronic infection that can last for several decades (*3**,**4*).

Immunocompetent persons are usually asymptomatic and periodically exhibit eosinophilia. In immunocompromised patients, the endogenous autoinfection cycle may result in the overproduction and dissemination of larvae into intestinal and extraintestinal tissues, including the central nervous system, leading to the hyperinfection syndrome which can be lethal (*5*). Most cases (96%) occur in immigrants, but some have been described in patients with a history of travel, sometimes many years previously. *S. stercoralis* infections have been reported up to 65 years after initial exposure in veterans who served in Asia during World War II (*4**,**6*).

Although our patient exhibited poor general condition, he likely did not experience hyperinfection syndrome because he was not immunosuppressed, and he completely recovered after receiving standard ivermectin treatment. That the patient was originally infected in the Canary Islands seems improbable, although a low level of transmission exists in rural and disadvantaged areas in continental Spain, Portugal, and Italy (*7*). We did not find evidence of *Strongyloides* spp. transmission in the Canary Islands. In particular, the patient stayed in a high-status tourist hotel for a short period, and he never walked in bare feet. He was probably infected when he lived in Vietnam.

This case highlights the importance of systematically considering chronic strongyloidiasis when seeking a diagnosis for persistent hypereosinophilia, even in patients with no underlying disease, and the value of systematically obtaining any history of travel in disease-endemic areas even if it occurred many years previously. The endogenous autoinfection cycle can possibly persist for a lifetime. In addition, systematic examination of stool samples should be carried out, and ivermectin should be given when an immunosuppressive drug is required in a patient who has a history of travel to, or residence in, an area to which strongyloidiasis is endemic.
